# Association of Thiazide Use in Patients with Hypertension with Overall Fracture Risk: A Population-Based Cohort Study

**DOI:** 10.3390/jcm11123304

**Published:** 2022-06-09

**Authors:** Cheng-Hsun Chuang, Shun-Fa Yang, Pei-Lun Liao, Jing-Yang Huang, Man-Yee Chan, Chao-Bin Yeh

**Affiliations:** 1 Institute of Medicine, Chung Shan Medical University, Taichung 402, Taiwan; skdef37372@hotmail.com.tw (C.-H.C.); ysf@csmu.edu.tw (S.-F.Y.); wchinyang@gmail.com (J.-Y.H.); 2 Department of Emergency Medicine, School of Medicine, Chung Shan Medical University, Taichung 402, Taiwan; 3 Department of Emergency Medicine, Chung Shan Medical University Hospital, Taichung 402, Taiwan; 4 Department of Medical Research, Chung Shan Medical University Hospital, Taichung 402, Taiwan; liaopeilun0410@gmail.com; 5 Department of Dentistry, Taichung Tzu Chi Hospital, Buddhist Tzu Chi Medical Foundation, Taichung 427, Taiwan

**Keywords:** thiazide, hypertension, risk of fracture

## Abstract

Thiazide diuretics have long been widely used as antihypertensive agents. In addition to reducing blood pressure, thiazides also control calcium homeostasis and increase bone density. We hypothesized that the use of thiazides in patients with hypertension would reduce overall fracture risk. We used the Taiwan National Health Insurance Research Database to find patients with a hypertension diagnosis who accepted antihypertensive treatment from 2000 to 2017. The patients were further classified into thiazide users and nonthiazide users. Multivariable Cox regression analysis and Kaplan–Meier survival analysis were performed to estimate the adjusted hazard ratios (aHRs) and cumulative probability of fractures. After 1:1 propensity score matching by sex, age, urbanization level of place of residence, income, comorbidities, and medications, there were 18,483 paired thiazide users and non-users, respectively. The incidence densities of fractures (per 1000 person-months) were 1.82 (95% CI: 1.76–1.89) and 1.99 (95% CI: 1.92–2.06) in the thiazide and nonthiazide groups, respectively. The results indicated a lower hazard ratio for fractures in thiazide users (aHR = 0.93, 95% CI: 0.88–0.98). Kaplan–Meier survival analysis revealed a significantly lower cumulative incidence of fractures in the thiazide group (log-rank test; *p* = 0.0012). In conclusion, our results reveal that thiazide use can reduce fracture risk. When antihypertensive agents are being considered, thiazide may be a better choice if the patient is at heightened risk of fracture.

## 1. Introduction

With an aging worldwide population, the importance of effectively treating muscle loss and osteoporosis is increasing. The characteristic of osteoporosis is bone mass loss and microarchitectural deterioration of bone tissue. Osteoporotic bone can easily fracture even in a minor collision [1], and osteoporotic fractures are one of the most common injuries encountered in the emergency department [2]. The most osteoporotic fracture locations were distal radius, proximal femur, and vertebral compression fractures. Calcium is the most abundant mineral in the body, 99% of which is found in the teeth and bones. Apart from bone, the two main organ systems responsible for calcium homeostasis are the intestines and kidneys [3,4,5]. Vitamin D improves the ability of the intestines to absorb calcium [6], and a calcium plus vitamin D supplement has been used to prevent osteoporotic fractures. A meta-analysis revealed a significant 15% reduction in the risk of total fractures and a 30% reduction in the risk of hip fractures when patients use calcium plus vitamin D supplementation for fracture prevention [7]. The kidneys play a key role in both calcium reabsorption and excretion. Approximately 200 mg of calcium per day is typically excreted by adults through the kidneys via urine [8], but this value varies by diet and serum parameters. Reducing the excretion of calcium from urine is one strategy to maintain adequate calcium in the human body [9].

In older people, hypertension is a common disease, and initial control through non-drug therapies, such as lifestyle modifications, body weight management, and increased exercise, is recommended [10,11]. Antihypertensive medications are used if non-drug therapy cannot achieve adequate blood pressure control. Many types of antihypertensive drugs have been developed. The four main classes of medications used in combination therapies for the treatment of hypertension are thiazide diuretics, calcium channel blockers (CCBs), angiotensin-converting enzyme inhibitors (ACEIs), and angiotensin receptor blockers (ARBs). The combination use of antihypertensive medications can achieve synergistic effects in blood pressure reduction with fewer doses [12,13,14]. Physicians select an antihypertensive treatment for a patient according to their underlying diseases or contraindications [15,16]. Patients with a reduced ejection fraction should initially be treated with a beta-blocker and an ACEI or ARB [16]. Patients with chronic kidney disease and proteinuria should be treated with an ACEI or ARB plus a thiazide diuretic or CCB [17]. If patients with diabetes mellitus have proteinuria, combination therapy should include an ACEI or ARB [18].

Thiazide diuretics have long been widely used as antihypertensive agents. Thiazides are defined as a third-line antihypertensive because they are less effective at reducing blood pressure than are ACEIs or ARBs. Thiazides inhibit the Na+/Cl cotransporter (NCC) in the convoluted renal distal tubule. The NCC facilitates the reabsorption of sodium from the distal tubules to the interstitium. A decrease in sodium reabsorption results in an increase in urine output, leading to a decrease in plasma volume and decreased blood pressure.

In addition to reducing blood pressure, thiazides control calcium homeostasis and increase bone density. Thiazides reduce urinary calcium excretion and stimulate osteoblast differentiation and bone mineral formation [7]. The mechanism by which thiazides reduce calcium excretion remains unclear. In some studies, thiazide has been used in idiopathic hypercalciuria [19]. Li et al. reported that the long-term use of thiazide diuretics reduces the incidence of recurrent renal calculi and the 24-h urinary calcium level [20]. By reducing calcium loss, the bone density and calcium within bones increase. Aung et al. reported that thiazide could reduce the incidence of hip fractures [21].

In perimenopausal or postmenopausal women, osteoporotic fractures are one of the common complications [22]. Several therapies have been proposed to prevent them. Calcium plus vitamin D, hormone therapy, or combination therapy are the current primary treatments to protect against osteoporotic fractures [23].

Our primary outcome of the study was the fracture rate of the hypertensive participant and the adjusted hazard ratio between the thiazide user and the non-user for hypertension using data from the Taiwan National Health Research Database of Taiwan (NHIRD). Our second objective was to determine whether thiazide can prevent fractures in perimenopausal or postmenopausal women.

## 2. Materials and Methods

### 2.1. Study Design and Population

As stated, we used data from Taiwan’s NHIRD; Taiwan implemented National Health Insurance (NHI) in 1995, and the database contains NHI claims data for more than 99% of Taiwan’s population and provides a means to explore the risk factors or effects of disease interventions. This study used a retrospective cohort design with NHIRD data from 2000 to 2017. Diseases were diagnosed using the International Classification of Diseases, Ninth [Tenth] Edition, Clinical Modification (ICD-9(10)--CM). This study was approved by the Institutional Review Board of Chung Shan Medical University Hospital (approval number CS2-20036).

### 2.2. Study Population

Records from 2000 to 2017 were collected from the NHIRD, and the study population included patients with hypertension (ICD-9-CM codes 401-405 and ICD-10-CM codes I10-I15). Patients must have used a hypertension medication within 1 year of diagnosis, including beta-blockers (anatomic therapeutic chemical [ATC] code: C07), CCBs (ATC code: C08), alpha-blockers (ATC code: C02CA), and ACEIs or ARBs (ATC code: C09).

We identified a total of 498,738 patients with hypertension. After accounting for the excluded conditions, we finally included 216,867 patients for data analysis. We divided patients into two groups: hypertension with thiazide use (HT-with-thiazide) and hypertension without thiazide use (HT-without-thiazide). A total of 18,620 patients were placed in the HT-without-thiazide group and 198,247 patients were placed in the HT-without-thiazide group. The index date was defined as the first day 365 days after the diagnosis of hypertension. This setting is because we defined patients who continuously used thiazide for 1 year as stable users and assumed that these stable users would continue to use thiazide during follow-up.

### 2.3. Characteristics, Comorbidities, and Outcomes

We identified baseline demographic characteristics (as reported within 365 days before the index date), such as age and sex, and the comorbidities and medications of each participant to evaluate their health status. Baseline comorbidities included diabetes mellitus, hyperlipidemia, ischemic heart disease, cerebrovascular accident, abnormal renal function, chronic obstructive pulmonary disease (COPD), cancer, and depressive disorders. Baseline medications included beta-blockers, CCBs, ACEIs, ARBs, corticosteroids, non-steroidal anti-inflammatory drugs (NSAIDs), proton-pump inhibitors, and hormonal medications.

The study outcome was defined as the diagnosis of a fracture, differentiated into skull, spine and trunk, upper limb, lower limb, and pathological fractures. All study individuals were followed up from the index date to either the study outcome, the occurrence of fracture due to car accident, death, or the end of the study (31 December 2017).

### 2.4. Statistical Analysis

Statistical analysis was performed using SAS version 9.4 (SAS Institute, Cary, NC, USA), and a *p*-value of <0.05 was considered statistically significant. HT-with-thiazide patients were matched with HT-without-thiazide patients by age (±1 year) and sex at a 1:4 ratio. To reduce potential confounding bias due to measured factors, 1:1 propensity score matching (PSM) was performed by using the greedy nearest neighbor algorithm and noreplacement matching with a caliper width of 0.01; matched variables included birth year, sex, age (±1 year) at the index date, index year, comorbidities, and medication. The absolute standardized difference (ASD) was used to evaluate the differences in covariates between the two study groups; an ASD value of <0.1 indicated that the item was balanced between the groups.

Categorical data were presented as numbers and percentages, and the differences in categorical variables were compared using the chi-square test. Incidence rates with corresponding confidence intervals (CI) and crude hazard ratios (HRs) were calculated using Poisson regression. After the proportional hazards assumption was tested, a Cox proportional hazards model analysis was performed to estimate the HRs for mortality and 95% CIs. Cumulative fracture probabilities were assessed using Kaplan–Meier analysis, in which statistical significance was determined using the results of a log-rank test.

## 3. Results

### 3.1. Characteristics of the Participants

**Figure 1:**
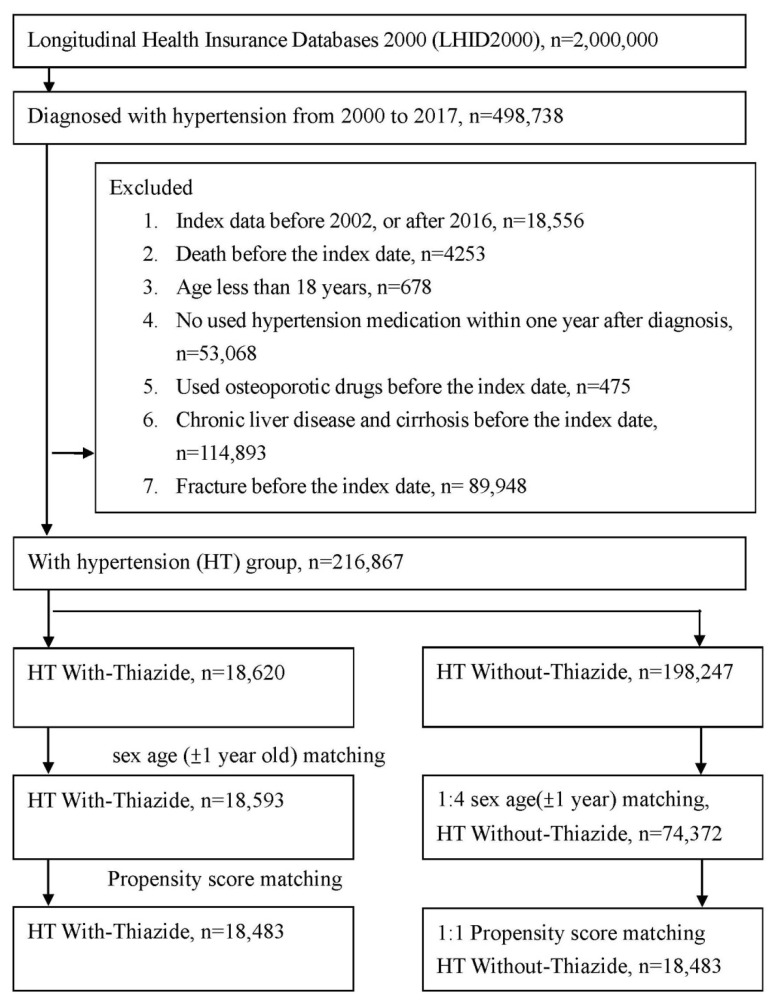
Study flowchart of patient selection.

The selection flow chart for this study is presented in Figure 1. A total of 18,593 patients were included in the HT-with-thiazide group, and an additional 74,372 patients were sex and age (±1 years) matched at a 1:4 ratio to form the HT-without-thiazide control group (Supplementary Table S1). After 1:1 PSM matching by sex, age, urbanization level of place of residence, income, comorbidities, and medications, 18,483 HT with-thiazide and HT without-thiazide participants were obtained; 58% of the patients were male, and 41% were female. Over 57% were aged 46 to 60 years. The baseline characteristics are presented in Table 1.

### 3.2. Risk of Fracture between HT-with-Thiazide and HT-without Thiazide Group

**Figure 2:**
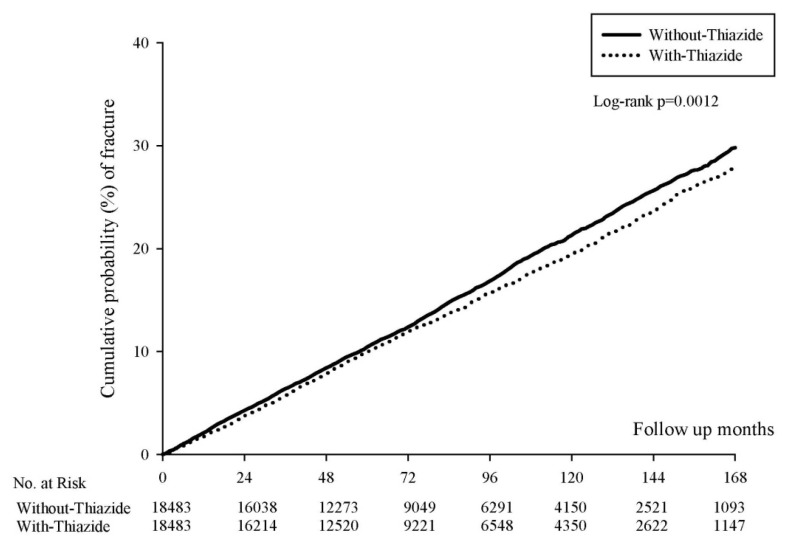
Kaplan–Meier curves of the cumulative proportions of fracture in the use of thiazide and without the use of thiazide.

As Table 2 indicates, the incidence densities of fractures (per 1000 person-months) were 1.83 (95% CI: 1.76–1.90) and 1.97 (95% CI: 1.94–2.01) in the sex- and age-matched HT-with-thiazide and HT-without-thiazide cohorts, respectively (Supplementary Table S2). After PSM, the values were 1.82 (95% CI: 1.76–1.89) and 1.99 (95% CI: 1.92–2.06) in the HT-with-thiazide and HT-without thiazide cohorts, respectively, and the aHR for the HR-with-thiazide group was 0.93 (95% CI: 0.88–0.98). Kaplan–Meier survival analysis revealed a significantly lower cumulative incidence of fractures in the HT-with-thiazide group (log-rank test; *p* = 0.0012; Figure 2).

Multiple cox regression revealed that the aHR of fractures for the HT-with-thiazide group was significantly lower than that of the HT-without-thiazide group. Other significant risk factors for fractures were sex; age; urbanization level; income; the comorbidities of diabetes mellitus, hyperlipidemia, cerebrovascular accident, abnormal renal function, and COPD; and the medications of corticosteroids and NSAIDs (Supplementary Table S3).

**Figure 3:**
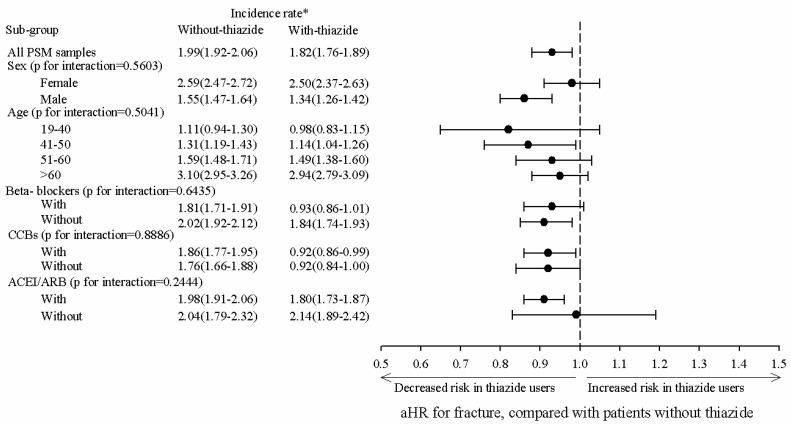
Subgroup analysis of adjusted hazard ratio for fractures in patients with thiazide compared with patients without thiazide. * per 1000 person-months; aHR (adjusted Hazard Ratio) adjusted variables including age, sex, comorbidities and medication; CCBs: Calcium Channel Blockers; ACEIs: Angiotensin-Converting Enzyme Inhibitors; ARB: Angiotensin Receptor Blockers.

In Figure 3, subgroup analysis revealed that the aHR for women and men was 0.98 (95% CI: 0.91–1.05) and 0.86 (95% CI: 0.80–0.93), respectively, and the HR-with-thiazide group exhibited a significantly reduced risk compared to the HR-without-thiazide group. For the age 41 to 50 years group, the aHR was 0.87 (95% CI: 0.76–0.99). For the with-CCBs group, the aHR was 0.92 (95% CI: 0.86–0.90). For the with-ACEI or ARBs group, the aHR was 0.91 (95% CI: 0.86–0.96).

## 4. Discussion

Hypertension is a common chronic disease among older adults. Several hypotheses accounting for the pathophysiology have been proposed. One of the hypotheses concerns maladaptation to a high-salt diet. Hypertension may be a physiological response intended to excrete excess salt. Natriuresis is a key treatment for hypertension [24]. Diuretics could remove excessive salt to achieve hypertension control. A review concluded that the use of a low-dose thiazide reduced all mortality and morbidity in adult patients with moderate-to-severe primary hypertension [25].

Older people are also more susceptible to osteoporotic fractures and even low-energy injuries due to low bone density and being prone to falls. Hip fracture is also one of the risks associated with higher mortality and morbidity among older adults. The cumulative mortality rate for 12 months was 33%, and the 1-year mortality rate increased significantly by 2% per year [26] Several cost-effective pharmacologic treatments may be used to prevent fractures, such as vitamin D and calcium supplements and bisphosphonates [27]. Denosumab is an effective option for preventing fracture, which reduces bone resorption to achieve bone mass preservation [28].

Our study aimed to determine whether thiazide could be used to reduce fracture risk in patients with hypertension. This has remained controversial in prior research. A meta-analysis determined that the use of thiazide was associated with reduced risk in case-control studies but not in cohort studies. That study concluded that the use of thiazide might not protect against fractures [29]. However, some studies have different conclusions. A recent study using a Swedish database concluded that the use of bendroflumethiazide or hydrochlorothiazide could reduce the risk of hip fractures [30]. That study also noted that the choice of antihypertensive can influence fracture risk. A recent meta-analysis including 22 observational studies concluded the use of thiazide was associated with a lower fracture rate of the hip [31]. A meta-analysis that compared patients with diuresis with and without thiazide determined that thiazide reduced overall fracture risk by 14% and hip fracture risk by 18% [32].

In the present population-based retrospective cohort study, we determined that patients with hypertension and thiazide had a lower overall fracture risk. The adjusted risk ratio for the HT-with-thiazide group was 0.926, indicating thiazide has a protective effect against fracture for patient hypertension.

A meta-analysis found the protective effect on fracture risk is associated with the long duration and continuity of thiazide use [33]. Similar to our study, the protective effect seems more significant after 72 months from Kaplan–Meier curves (Figure 2). Another meta-analysis concluded that the association between the use of thiazide and risk of osteoporotic fracture is not significant. However, this study also concluded that the different general status of a patient might have different levels of benefit [34]. This study gave us an important implication that we should find the specific population that could benefit from thiazide diuretics for preventing fracture. In our study, subgroup analysis was used to determine whether thiazide could protect against fractures in perimenopausal and postmenopausal women.

A retrospective cohort study using the MJ Health Database in Taiwan indicated that the mean age at menopause for women in Taiwan is 50.2 [35]. The adjusted fracture risk ratio was 0.88 in a subgroup of females aged 41–50. (95% C.I. 0.73–1.06) The possible explanation is sample size was not large enough to meet statistical significance. The adjusted fracture risk ratio was 0.87 in a group aged 41–50. (95% C.I. 0.76–0.99) A possible explanation for this is as follows: As people reach their forties and start to lose protection from hormones, thiazide can compensate to improve calcium reabsorption and increase bone density. However, during their fifth decade or older, people completely lose the protection of hormones. Bone mineral loss accelerates and worsens, and thiazide does not provide sufficient protection.

Bone mineral density (BMD) measurements provide a snapshot of bone health, including the presence of osteoporosis and the risk of fractures. Two randomized controlled trials revealed that thiazide achieved significant benefits in BMD in postmenopausal women [36,37]. To sum up, thiazide could produce a positive effect on BMD, but increased BMD does not necessarily indicate reduced fracture risk.

A meta-analysis revealed that the risk of osteoporotic fractures among individuals with hypertension was higher than individuals without hypertension, with an odds ratio of 1.33 [38]. A longitudinal study indicated that hypertension is an independent risk factor for fractures in women but not men [39]. Although our study observed that thiazide had a protective effect on fracture in patients with hypertension, we were unable to determine whether the protective effect originates from the control of blood pressure or thiazide facilitating calcium reabsorption.

The most common indication for thiazide is hypertension; others include edema or ascites secondary to cirrhosis or heart failure. In several studies, the research participants were not specifically individuals with hypertension [21,29,40]. Different from those studies, the participants in our study used thiazide specific for hypertension.

Our study has several limitations. First, the claims records in the NHIRD are mainly used to calculate medical unit services and service costs. Several key indicators recorded in clinical practice are not included in the database, such as the severity, type, and mechanism of patient fractures. We used PSM to eliminate possible confounding factors that were recorded in the database. Second, the retrospective cohort design of this study excluded causal inference and limited the precision of our study. Therefore, we matched the study group with the control group by propensity score to reduce this bias. Third, BMD is a useful indicator of bone health. A previous study concluded that thiazide could improve BMD [41]. However, BMD values are not recorded in the NHIRD. Further cohort studies or studies using hospital electronic medical records systems might help to clarify the relationships between BMD and the use of thiazide.

## 5. Conclusions

In conclusion, our study revealed that thiazide use for hypertension can reduce overall fracture risk. When deciding between antihypertensive agents, thiazide may be a favorable choice if the patient is at elevated risk of fragility fractures. Through subgroup analysis, we also determined that women over 40 years of age who used thiazide were at a reduced risk of fractures. This could be an indication for the use of antihypertensive medications in perimenopausal women.

## Figures and Tables

**Table 1:** Baseline characteristics among study groups.

	After PSM		
Variables	Without-Thiazide n = 18,483	With-Thiazide n = 18,483	ASD
Index year			0.0244
2002–2006	5573 (30.15%)	5660 (30.62%)	
2007–2011	5831 (31.55%)	5842 (31.61%)	
2012–2016	7079 (38.3%)	6981 (37.77%)	
Sex			0.0020
Female	7689 (41.6%)	7707 (41.7%)	
Male	10,794 (58.4%)	10,776 (58.3%)	
Age at index			0.0000
19–45	3463 (18.74%)	3543 (19.17%)	
46–60	10,616 (57.44%)	10,544 (57.05%)	
≥61	4404 (23.83%)	4396 (23.78%)	
Urbanization			0.0412
Urban	11,580 (62.65%)	11,534 (62.4%)	
Sub-urban	5731 (31.01%)	5753 (31.13%)	
Rural	1172 (6.34%)	1196 (6.47%)	
Income			0.0061
1–22,000	6218 (33.64%)	6271 (33.93%)	
>22,000	12,265 (66.36%)	12,212 (66.07%)	
Comorbidities			
Diabetes mellitus	5530 (29.92%)	5493 (29.72%)	0.0044
Hyperlipidemia	7209 (39%)	7124 (38.54%)	0.0094
Ischemic heart disease	3281 (17.75%)	3302 (17.87%)	0.0030
Cerebrovascular accident	2198 (11.89%)	2297 (12.43%)	0.0164
Abnormal renal function	1140 (6.17%)	1199 (6.49%)	0.0131
COPD	1132 (6.12%)	1210 (6.55%)	0.0173
Cancer	482 (2.61%)	575 (3.11%)	0.0302
Depressive disorders	459 (2.48%)	508 (2.75%)	0.0166
Medication			
Beta- blockers	8035 (43.47%)	7934 (42.93%)	0.0110
CCBs	10,960 (59.3%)	10,928 (59.12%)	0.0035
Alpha-blockers	880 (4.76%)	979 (5.3%)	0.0245
ACEI/ARB	17,326 (93.74%)	17,326 (93.74%)	0.0000
corticosteroids	9427 (51%)	9460 (51.18%)	0.0036
NSAIDs	12,540 (67.85%)	12,402 (67.1%)	0.0159
PPIs	989 (5.35%)	1087 (5.88%)	0.0230
Hormonal medications	818 (4.43%)	859 (4.65%)	0.0107

ASD: Absolute Standardized Difference; COPD: Chronic Obstructive Pulmonary Disease; CCBs: Calcium Channel Blockers; ACEIs: Angiotensin-Converting Enzyme Inhibitors; ARB: Angiotensin Receptor Blockers; NSAIDs: Non-Steroidal Anti-Inflammatory Drugs; PPIs: Proton Pump Inhibitors.

**Table 2:** Incidence density of fracture.

	After PSM	
Variables	Without-Thiazide	With-Thiazide
Number	18,483	18,483
Follow up person months	1,428,347	1,457,827
New fracture case *	2848	2666
Incidence rate * (95% C.I.)	1.99 (1.92–2.06)	1.82 (1.76–1.89)
Crude Relative risk (95% C.I.)	Reference	0.92 (0.87–0.97)
Adjusted hazard ratio ^†^ (95% C.I.)	Reference	0.93 (0.88–0.98)
Competing Risk (95% C.I.)	Reference	0.93 (0.88–0.98)

* per 1000 person-months. ^†^ Adjusted variables including age, sex, comorbidities and medication.

## Data Availability

Restrictions apply to the availability of these data. Data were obtained from the National Health Insurance database and are available from the authors with the permission of the National Health Insurance Administration of Taiwan.

## Supplementary Materials

The following supporting information can be downloaded at: https://www.mdpi.com/article/10.3390/jcm11123304/s1, Table S1: Baseline characteristics among study groups. Table S2: Incidence density of fracture. Table S3: Multiple Cox proportional hazard regression results for fracture.
