# Supplementary material for: Association of Thiazide Use in Patients with Hypertension with Overall Fracture Risk: A Population-Based Cohort Study
Source: J Clin Med. 2022 Jun 9;11(12):3304. doi: 10.3390/jcm11123304 (PMC9225296; doi:10.3390/jcm11123304)
Supplement: Supplementary file 1 [file jcm-11-03304-s001.zip › jcm-1721624-supplementary.pdf]

Supplementary Table S1. Baseline characteristics among study groups.

Variables	Before PSM		
	**Without-Thiazide n=74372**	**With-Thiazide n=18593**	**ASD**
Index year			0.0000
2002-2006	22796(30.65%)	5699(30.65%)	
2007-2011	23560(31.68%)	5890(31.68%)	
2012-2016	28016(37.67%)	7004(37.67%)	
Sex			0.0000
Female	31084(41.8%)	7771(41.8%)	
Male	43288(58.2%)	10822(58.2%)	
Age at index			0.0000
19-45	14314(19.25%)	3575(19.23%)	
46-60	33989(45.7%)	8520(45.82%)	
>=61	26069(35.05%)	6498(34.95%)	
Urbanization			0.1445
Urban	42424(57.04%)	11627(62.53%)	
Sub-urban	25483(34.26%)	5769(31.03%)	
Rural	6465(8.69%)	1197(6.44%)	
Income			0.0145
1-22000	24716(33.23%)	6306(33.92%)	
>22000	49656(66.77%)	12287(66.08%)	
Co-morbidities			
Diabetes mellitus	11660(15.68%)	5593(30.08%)	0.3480
Hyperlipidemia	17920(24.1%)	7223(38.85%)	0.3218
Ischemic heart disease	10670(14.35%)	3325(17.88%)	0.0963
Cerebrovascular accident	5959(8.01%)	2348(12.63%)	0.1522
Abnormal renal function	2470(3.32%)	1253(6.74%)	0.1569
COPD	5058(6.8%)	1215(6.53%)	0.0107
Cancer	2677(3.6%)	577(3.1%)	0.0276
Depressive disorders	2532(3.4%)	509(2.74%)	0.0387
Medication			
Beta- blockers	32375(43.53%)	8022(43.15%)	0.0078
CCBs	48631(65.39%)	11024(59.29%)	0.1261
Alpha-blockers	3177(4.27%)	1004(5.4%)	0.0526
ACEI/ARB	34649(46.59%)	17435(93.77%)	1.2038
corticosteroids	39149(52.64%)	9518(51.19%)	0.0290
NSAIDs	54997(73.95%)	12437(66.89%)	0.1551
PPIs	4397(5.91%)	1093(5.88%)	0.0014
Hormonal medications	4004(5.38%)	861(4.63%)	0.0345

ASD: Absolute Standardized Difference.; COPD: Chronic Obstructive Pulmonary Disease.; CCBs: Calcium Channel Blockers.; ACEIs: Angiotensin- Converting Enzyme Inhibitors.; ARB: Angiotensin Receptor Blockers.; NSAIDs：Non-Steroidal Anti-Inflammatory Drugs.; PPIs: Proton Pump Inhibitors.

Supplementary Table S2. Incidence density of fracture.

Variables	Before PSM	
	**Without-Thiazide**	**With-Thiazide**
N	74372	18593
Follow up person months	5880671	1465786
New fracture case*	11633	2685
Incidence rate*(95% C.I.)	1.97(1.94-2.01)	1.83(1.76-1.90)
Crude Relative risk (95% C.I.)	Reference	0.93(0.89-0.97)
Adjusted hazard ratio† (95% C.I.)	Reference	0.94(0.90-0.98)
Competing Risk (95% C.I.)	Reference	0.94(0.90-0.98)

*per 1000 person-months.

† Adjusted variables including age, sex, comorbidities and medication.

Supplementary Table S3. **Multiple Cox proportional hazard regression results for fracture.**

Variable	aHR (95% C.I.) Before PSM	After PSM
Exposure (ref: without-Thiazide)		
With-Thiazide	0.94(0.90-0.98)	0.93(0.88-0.98)
Index year (ref: 2002-2006)		
2007-2011	0.91(0.87-0.94)	0.88(0.82-0.93)
2012-2016	0.90(0.85-0.95)	0.93(0.85-1.01)
Sex (ref: Female)		
Male	0.65(0.62-0.67)	0.61(0.57-0.63)
Age at index (ref: 19-45)		
46-60	1.25(1.18-1.32)	1.31(1.20-1.42)
>=61	2.36(2.23-2.49)	2.62(2.39-2.87)
Urbanization (ref: Urban)		
Sub-urban	1.09(1.05-1.13)	1.11(1.04-1.17)
Rural	1.23(1.16-1.29)	1.24(1.12-1.36)
Income (ref: 1-22000)		
>22000	0.88(0.85-0.91)	0.91(0.85-0.95)
Co-morbidities (ref: non)		
Diabetes mellitus	1.22(1.16-1.27)	1.23(1.16-1.30)
Hyperlipidemia	0.93(0.89-0.96)	0.94(0.88-0.99)
Ischemic heart disease	1.04(1.00-1.09)	1.04(0.97-1.11)
Cerebrovascular accident	1.14(1.07-1.19)	1.12(1.03-1.20)
Abnormal renal function	1.21(1.11-1.31)	1.19(1.07-1.32)
COPD	1.24(1.16-1.30)	1.21(1.10-1.33)
Cancer	1.08(0.98-1.18)	1.12(0.96-1.30)
Depressive disorders	1.25(1.15-1.35)	1.14(0.97-1.32)
Medication (ref: non)		
Beta- blockers	0.95(0.92-0.98)	0.96(0.90-1.01)
CCBs	1.05(1.01-1.09)	1.03(0.97-1.08)
Alpha-blockers	1.00(0.92-1.08)	0.98(0.86-1.10)
ACEI/ARB	0.99(0.95-1.02)	0.97(0.87-1.06)
corticosteroids	1.14(1.09-1.17)	1.14(1.08-1.20)
NSAIDs	1.21(1.16-1.26)	1.19(1.12-1.26)
PPIs	1.08(1.00-1.16)	1.08(0.96-1.21)
Hormonal medications	0.99(0.92-1.05)	0.95(0.85-1.06)
